# New Approach Methodologies (NAMs) in Alternative Methods: A Comparative Cytotoxicity Analysis of Nanostructured Hydroxyapatite in Adipose Stem Cells Spheroids

**DOI:** 10.1002/jat.70057

**Published:** 2026-01-29

**Authors:** Bianca Montenegro, Rosângela Mayer Gonçalves, Marcelle Gomes Pegurier, Isabelle Amorim, Alexandre Malta Rossi, Leandra Santos Baptista

**Affiliations:** ^1^ Laboratório de Biologia de Células Eucarióticas, Instituto Nacional de Metrologia, Qualidade e Tecnologia Duque de Caxias Rio de Janeiro Brazil; ^2^ Programa de Pós‐Graduação em Biomedicina Translacional Duque de Caxias Rio de Janeiro Brazil; ^3^ Departamento de Matéria Condensada, Física Aplicada e Nanociência Centro Brasileiro de Pesquisas Físicas Rio de Janeiro Brazil; ^4^ Universidade Federal do Rio de Janeiro, Campus UFRJ Duque de Caxias Prof Geraldo Cidade Rio de Janeiro Brazil

**Keywords:** adipose stem cells, hydroxyapatite, nanoparticles, nanotoxicology, new approach methodologies, spheroids

## Abstract

Toxicological assessment is essential in NP approval for health and medical applications. Although 2D cell culture has been widely used, 3D models, especially spheroids, provide better predictive value for toxicological risk assessments since they replicate complex cellular interactions more accurately. In this study, different cytotoxicity assays were used to conduct a comparative nanotoxicological analysis of Adipose Stem Cells (ASC) grown in 2D and 3D (spheroid) systems, based on OECD guidelines. To evaluate the toxicity of nanostructured carbonated hydroxyapatite (nCHA) in ASC spheroids, the Neutral Red Uptake assay, and an ATP quantification assay were used. The results obtained from 2D cell monolayers and 3D spheroids were compared to determine the most suitable assay for spheroid analysis. Moreover, the IC50 for SDS treatment was determined and the spheroid morphology was analyzed after treatment with the nanoparticles. Overall, the three assays confirmed the absence of cytotoxicity of nCHA NPs in the ASC monolayer. In addition, the ATP quantification assay confirmed the absence of cytotoxicity of nCHA NPs in the ASC spheroids. Curiously, SDS cytotoxicity was higher in spheroids than in monolayer cultures, inducing the disaggregation of spheroids in a facile and concentration‐dependent manner (IC₅₀ = 9.67 μg/mL in spheroids, approximately eight times lower than the IC₅₀ observed in monolayer cultures, 76 μg/mL). The nCHA NPs were also explored as spheroid functionalization agents. The nanoparticles did not affect ASC spheroids' morphology and diameter, and the nCHA are located mainly in the extracellular matrix of spheroids mimicking the mineral component of bone. Collectively, our findings demonstrated that spheroids are more sensitive than monolayers for evaluating nanoparticle biocompatibility, highlighting the potential of 3D cultures as new advanced models (NAMs) for improved nanotoxicology assessments. Furthermore, the functionalization of ASC spheroids with nCHA NP holds potential both as a 3D osteogenesis model and as a therapeutic product to promote bone regeneration.

## Abstract 2

The study compared nCHA cytotoxicity in ASC cultured in 2D and 3D spheroids. Neutral Red, ATP assays, and SDS IC50 showed no cytotoxicity of the nanoparticles in either model. SDS was more toxic in spheroids, causing concentration‑dependent disaggregation. nCHA did not alter spheroid morphology and localized mainly in the extracellular matrix. Overall, spheroids were more sensitive for assessing nanoparticle biocompatibility, and nCHA‑functionalized spheroids show potential for 3D osteogenesis modeling and bone regeneration applications.

**Abbreviations**
ASC: adipose‐derived stem cell
ATP: adenosine triphosphate
CHA: carbonated hydroxyapatite
DMEM: Dulbecco's Modified Eagle Medium
ECM: extracellular matrix
EDS: energy‐dispersive x‐ray spectroscopy
EDTA: ethylenediaminetetraacetic acid
EELS: electron energy loss spectroscopy
FTIR: Fourier transform infrared spectroscopy
HA: hydroxyapatite
HR‐TEM: high‐resolution transmission electron microscopy
ITS: insulin–transferrin–selenium
nCHA: nanostructured carbonated hydroxyapatite
NP: nanoparticle
OECD: Organisation for Economic Co‐operation and Development
PBS: phosphate‐buffered saline
SAED: selected area electron diffraction
SDS: sodium dodecyl sulfate
TEM: transmission electron microscopy
NRU: neutral red uptake
XRD: X‐ray diffraction
XRF: X‐ray fluorescence

## 1 Introduction

Spheroids have proven valuable in tissue engineering and regenerative medicine as scaffold‐free approaches or as building blocks for bioprinting (Chae et al. 2021). Recently, the use of spheroids and biomaterials together to enhance their applications has been proposed (Caprio and Burdick 2023). The incorporation of biomaterials such as nanoparticles into spheroids can be used to guide their formation and differentiation while also serving as a functionalization strategy to enhance their applications in tissue engineering (Caprio and Burdick 2023). For example, adding hydroxyapatite nanoparticles to adipose stem cell (ASC) spheroids can boost their osteogenic potential (Wolff et al. 2023), effectively mimicking mineralized bone tissue (Kronemberger et al. 2021). This approach has significant implications for bone engineering. Moreover, incorporating microparticles or nanoparticles into spheroids has emerged as a strategy to deliver growth factors locally, overcoming the diffusion restrictions of these molecules from the culture media (Caprio and Burdick 2023). This strategy is commonly used in bone and cartilage tissue engineering, especially for delivering bone morphogenetic proteins (BMPs) and transforming growth factor beta (TGF*β*). For example, Solorio and collaborators used PLGA (poly (lactic‐co‐glycolic acid)) microparticles to deliver TGF‐*β*1, inducing chondrogenesis in mesenchymal stem cell spheroids (Solorio et al. 2010). Additionally, calcium phosphate‐based materials, such as hydroxyapatite, *β*‐tricalcium phosphate, and *β*‐glycerophosphate, have been incorporated into spheroids to stimulate bone repair due to their inherent osteoconductive properties (Zarkesh et al. 2019; Wang et al. 2016; Whitehead et al. 2019).

Although there are many advantages to the functionalization of mesenchymal stem cell spheroids, such as enhanced differentiation potential and secretory profile, there are important drawbacks to overcome (Kouroupis and Correa 2021). One of the most important is the compromise of viability potentially caused by the functionalizing biomaterial, creating necrotic cores and influencing spheroid size, nutrients, and oxygen concentration within the spheroid (Kouroupis and Correa 2021). The high surface area‐to‐volume ratio of nanoparticles enhances their reactivity and potential to interact with biological systems. While this property can be advantageous in therapeutic contexts, it also can potentially increase undesired or unpredictable biological responses, which may lead to cytotoxicity, oxidative stress, or inflammation (Sanità et al. 2020). At the nanoscale, hydroxyapatite (nHA) can be internalized by cells, potentially modulating metabolism and osteogenesis and exerting cytotoxic effects depending on the dose and exposure method (Zhang et al. 2017). Several studies have shown that nHA can be internalized by human umbilical cord Wharton's jelly‐derived mesenchymal stem cells (hWJ‐MSCs) and osteoblasts, promoting osteogenic differentiation by activating signaling pathways associated with matrix mineralization (Shi et al. 2018; Wang et al. 2019; Shi et al. 2009).

To the best of our knowledge, there are no published studies investigating nanoparticle toxicity in functionalized spheroids. Therefore, it is essential to evaluate the toxicity of nanoparticles to understand how the functionalization affects their potential use in regenerative medicine applications (Elmowafy et al. 2019). In this study, a comparative nanotoxicological analysis of nanostructured carbonated hydroxyapatite (nCHA) in both ASC monolayers (2D) and ASC spheroids (3D) was conducted based on the Organization for Economic Co‐operation and Development Guidelines for the Testing of Chemicals (OECD) guidelines. Furthermore, morphological parameters of ASC spheroids in the presence of nCHA were analyzed. This research underscores the potential of using spheroids as an alternative in vitro method to traditional 2D monolayer cultures, serving as a new approach methodology for nanotoxicology assessment and their potential functionalization for use in regenerative medicine applications.

## 2 Materials and Methods

### 2.1 Isolation and Culture of Human ASCs

Adipose‐derived stem cells (ASCs) were isolated from healthy human donors and cryopreserved as described by Baptista et al. (2009), according to the Research Ethics Committee of Clementino Fraga Filho University Hospital, Federal University of Rio de Janeiro, Brazil (Approval No. CAAE 25818719.4.0000.5257). ASCs were seeded into tissue culture flasks and maintained as described by Kronemberger et al. (2020). Summarily, ASC cell suspension at passage one was thawed and seeded into tissue culture flasks and maintained with TheraPEAK MSCGM‐CD, Mesenchymal Stem Cell Medium chemically defined (Lonza, São Paulo, Brazil) at 37°C in a humid atmosphere with 5% carbon dioxide (CO_2_). At monolayer confluence, cells were harvested using 0.125% trypsin (Gibco BRL, Rockville, MD, USA), with 0.78‐mM ethylenediaminetetraacetic acid (EDTA) (Invitrogen, São Paulo, Brazil). After reaching passage three, ASCs were used for spheroid or monolayer cultures in 96‐well plates. A single donor was used for all the experiments, and each experiment was performed three times.

### 2.2 Monolayer Culture

For monolayer cell culture, a total of 2 × 10^4^ ASCs were seeded into each well of 96‐well plates. Cells were cultivated in Dulbecco's Modified Eagle Medium (DMEM) low glucose supplemented with 5% fetal bovine serum (Sigma‐Aldrich, Munich, Germany), 60‐μg/mL penicillin, and 100‐mg/mL streptomycin.

### 2.3 Spheroid Culture

Micromolded non‐adhesive hydrogels with 81 circular recesses (2% of agarose—Ultrapure Agarose, Invitrogen, Waltham, MA, USA—in 0.9% NaCl) were prepared using silicone molds according to the manufacturer's recommendations (MicroTissues 3D Petri Dish, Sigma Aldrich, St. Louis, MO, USA). A total of 1 × 10^6^ ASCs were seeded into each micromolded non‐adhesive hydrogel recess in DMEM low glucose supplemented with InsulinTransferrin‐Selenium (ITS) (1×; Sigma Aldrich, St. Louis, MO, USA), 1.25‐μg/mL human albumin (Farma Biagini SPA, Castelvecchio Pascoli, Italy), 50‐μg/mL ascorbic acid (Sigma Aldrich, St. Louis, MO, USA), and 100‐μg/mL penicillin, 100‐μg/mL streptomycin (Sigma Aldrich, St. Louis, MO, USA) and maintained at 37°C and 5% CO_2_.

### 2.4 Synthesis of nCHA Sample

The nCHA sample was synthesized according to the same methods used by dos Anjos et al. (2019). Briefly, nCHA containing 0.8 wt% CO_3_ were synthesized by a wet precipitation method by adding (NH4)2HPO4 and (NH4)2CO_3_ solutions into Ca (NO_3_)2. 4H2O solution at 37°C and pH = 11. The Calcium and phosphate contents of the CHA samples were analyzed by X‐ray fluorescence (XRF) using a PW 2400 X‐Ray Spectrometer at 3.0 kV (Philips), and by vanadomolybdate phosphoric acid colourimetric method with a UV–Vis spectrophotometer at 420 nm (Shimadzu, UV‐2450), respectively. The phosphate for carbonate substitution was quantified in a SC‐144DR Sulfur and Carbon Analyser. The elemental chemical analyses were conducted in triplicate. The surface area was assessed using the Brunauer, Emmett, and Teller (BET) technique with an ASAP 2020 Accelerated Surface Area and Porosimetry Analyser (Micromeritics, ASAP 2020 Accelerated).

### 2.5 Monolayer and Spheroid Culture Treatment With nCHA

Monolayer and spheroid cultures of ASCs were treated with nCHA at different concentrations (0.01, 0.1, 1, 10, 50, and 100 μg/mL). For monolayer cultures, nCHA was added when the cells reached approximately 70% confluence. For spheroid cultures, nCHA was associated with the ASCs immediately before spheroid formation, when the cells were trypsinized and resuspended in culture medium. A total of 1 × 10^6^ cells were plated per agarose micromold well, and spheroids were allowed to form in the presence of nCHA without subsequent replenishment. Sodium dodecyl sulfate (SDS) (Sigma Aldrich, St. Louis, MO, USA) was used as a positive control and added to the culture media at the following concentrations: 6.8, 10, 14.7, 21.5, 31.6, 46.4, 68.1, and 100 μg/mL. After 48 h, cell viability was assessed using neutral red uptake (NRU) and ATP quantification. The NRU assay was selected following OECD TG 432 guidelines (OECD 2019) due to potential nanoparticle interference with certain dyes (Mello et al. 2020; Andraos et al. 2020). An SDS standard curve in ASC monolayers was used as a positive control for cytotoxicity.

### 2.6 Monolayer: Cell Viability Assays

#### 2.6.1 Neutral Red Uptake (NRU) Assay

This assay was performed following the recommendations of the OECD (2019). In summary, after treatment with SDS or nCHA, cells were incubated for 3 h with neutral red dye (25 μg/mL) (Sigma Aldrich, St. Louis, MO, USA) dissolved in sterile water and maintained at 37°C in a 5% CO₂ atmosphere. Cells were then washed with phosphate‐buffered saline (PBS), and 100 μL of elution medium (EtOH:H₂O:acetic acid, 50:49:1) was added. The absorbance at 540 nm was measured using a microplate spectrophotometer system Synergy H4 Hybrid (BioTek, BioTek Instruments, Winooski, Vermont, EUA). The absorbance values were normalized to those of untreated cells, which served as the negative control and were considered to represent 100% viability. Results are presented as a percentage of these control values.

#### 2.6.2 ATP Quantification Assay

After treatment with SDS or nCHA, ATP quantification assay was performed using a commercially available kit (CellTiter‐Glo 2.0 Cell Viability Assay—Promega, Madison, USA) according to the manufacturer's recommendations. Briefly, 50 μL of culture medium remained in each well at the time of the assay, and an equal volume (50 μL) of detection reagent was added (v/v). The plate was homogenized for 2 min on an orbital shaker to induce cell lysis. Subsequently, it was allowed to rest for 10 min without light to stabilize the luminescence signal. Luminescence was measured using a microplate spectrophotometer system Synergy H4 Hybrid (BioTek, BioTek Instruments, Winooski, Vermont, EUA).

### 2.7 Spheroids: Cell Viability Assay

#### 2.7.1 ATP Quantification Assay

After treatment with SDS or nCHA, ATP quantification assay was performed using a commercially available kit (CellTiter‐Glo 3D, Promega, Madison, USA) according to the manufacturer's recommendations. Briefly, the spheroids in 50 μL of medium were transferred to a non‐adherent black 96‐well plate (Corning, Corning, NY, USA), 50 μL of the kit reagent was added to each well and vigorously homogenized for 5 min to induce cell lysis. Subsequently, the plate was incubated at room temperature and protected from light for 25 min to stabilize the luminescence signal. The luminescence was measured using a microplate spectrophotometer system Synergy H4 Hybrid (BioTek, BioTek Instruments, Winooski, Vermont, USA).

### 2.8 Spheroid Diameter Ratio

ASC spheroid images were acquired after 48 h of treatment with nCHA using an optical microscope (Primo Vert, Zeiss, USA) equipped with a digital camera. The width and length of each spheroid were measured using ZEISS ZEN lite software (Zeiss, Jena, Germany). To assess spheroid sphericity, the diameter ratio was calculated by dividing the width by the length (width/length). Values closer to 1.0 were interpreted as indicating more spherical and morphologically regular spheroids.

### 2.9 Transmission Electron Microscopy

For transmission electron microscopy (TEM) processing, spheroids associated with 50 μg/mL of nCHA were collected from the micromolded agarose hydrogel, which was then washed with 0.01 M phosphate‐buffered saline (PBS). The samples were fixed in 2.5% glutaraldehyde (Sigma Aldrich, St. Louis, MO, USA) and prepared in 0.1 M sodium cacodylate buffer (Sigma Aldrich, St. Louis, MO, USA). After fixation, the spheroids were rinsed with sodium cacodylate buffer and post‐fixed in 1% osmium tetroxide (Sigma Aldrich, St. Louis, MO, USA) diluted in 0.1 M sodium cacodylate buffer, with light protection. Subsequently, the samples underwent three additional washes with 0.1‐M sodium cacodylate buffer. Dehydration was carried out through a graded acetone series. Finally, the spheroids were embedded in Epon resin (Sigma Aldrich, St. Louis, MO, USA), diluted in acetone, and polymerized at 60°C for 48 h. Ultrathin sections (< 150 nm) of spheroids containing nCHA were prepared using an RMC PT‐XL PowerTome ultramicrotome. Imaging and analysis were performed using a high‐resolution transmission electron microscope (HR‐TEM) JEOL 2100F, equipped with a CCD camera, Energy‐Dispersive X‐ray Spectroscopy (EDS), Precession Electron Diffraction, Selected Area Electron Diffraction (SAED), and Electron Energy Loss Spectroscopy (EELS).

### 2.10 Statistical Analysis

All data are presented as mean ± standard deviation (SD) from three independent biological replicates (*n* = 3), each calculated as the arithmetic mean of six technical wells. Statistical analyses were performed in GraphPad Prism 8 (GraphPad Software, San Diego, CA, USA). Normality of the biological means was assessed using the Shapiro–Wilk test. For comparisons between two independent groups, the non‐parametric Mann–Whitney U test (unpaired, two‐tailed) was applied. For experiments involving multiple concentrations, one‐way ANOVA followed by Tukey's multiple comparisons test or the non‐parametric Kruskal–Wallis test was used, depending on data normality. Significance was considered at *α* = 0.05. For cytotoxicity dose–response analyses, IC₅₀ values were calculated by nonlinear regression using a four‐parameter logistic (4PL) model, and goodness‐of‐fit (*R* ^2^) and 95% confidence intervals were reported. Comparisons between curves were performed using the extra‐sum‐of‐squares *F* test. Raw absorbance or luminescence data were corrected by subtracting the mean background signal (medium and reagents without cells) and normalized to the mean of the untreated control group, which was set to 100% for ease of comparison.

## 3 Results

### 3.1 Compositional and Structural Analyses Validate the Synthesis of nCHA

**FIGURE 1:**
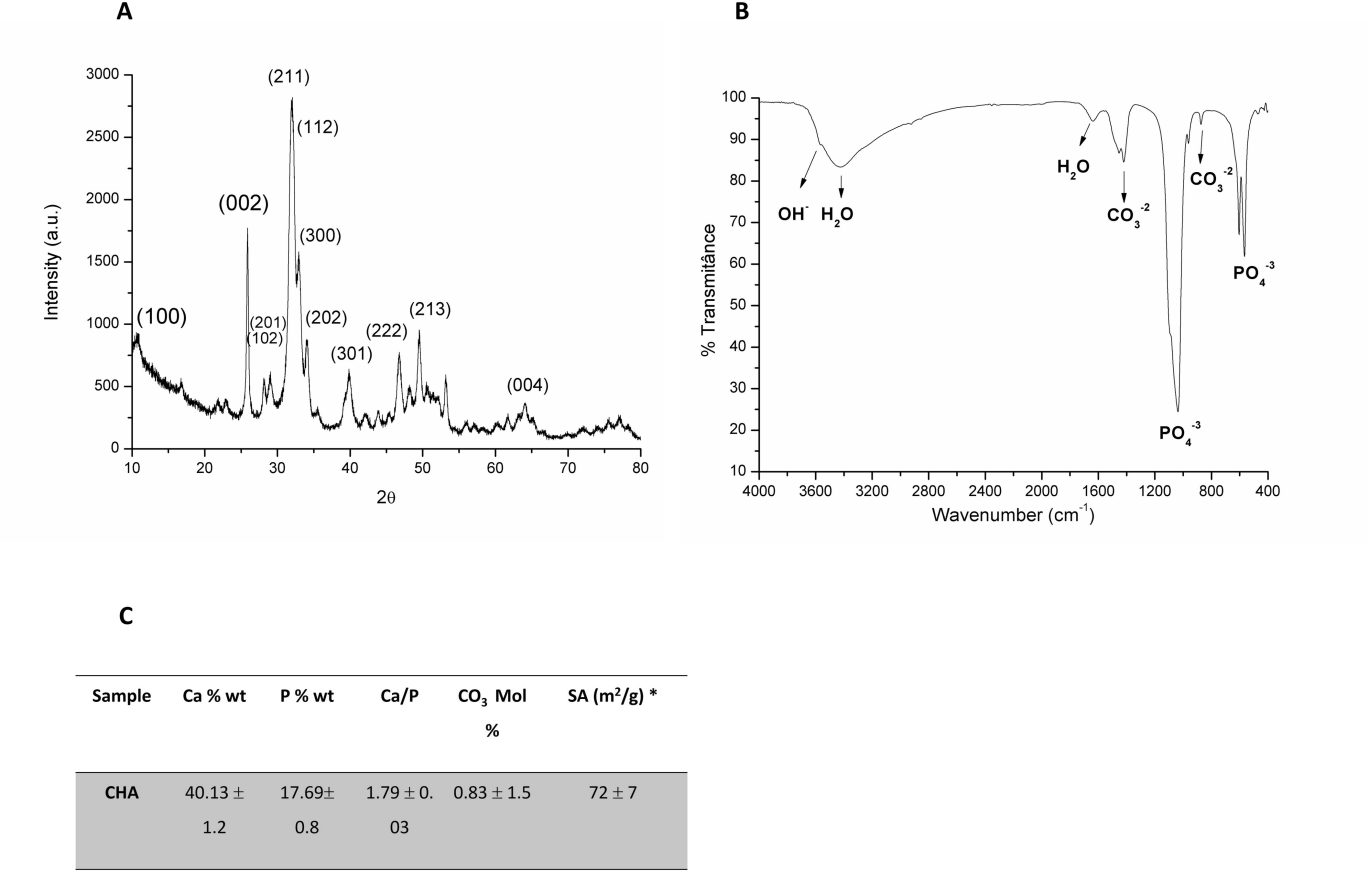
Characterization of nanostructured carbonated hydroxyapatite (nCHA) by XRD, FTIR, and elemental analysis. (A) The nCHA powder exhibits a typical X‐ray diffraction pattern of hydroxyapatite (HA), with broader peaks than those observed in stoichiometric HA, indicating carbonate‐substituted hydroxyapatite with low crystallinity. (B) The Fourier‐transform infrared (FTIR) spectrum shows vibrational bands in the 420‐ to 550‐cm^−1^ region, characteristic of CO₃^2−^ ions substituting phosphate groups in the HA lattice. (C) Elemental analysis confirmed the incorporation of 0.83 mol% CO₃^2−^ in the HA structure.

The synthesized nCHA was characterized using multiple analytical techniques to confirm its physicochemical properties. X‐ray diffraction (XRD) analysis revealed a typical hydroxyapatite (HA) pattern with broader peaks than those found in stoichiometric HA with a high degree of crystallinity, indicating the material's low crystallinity (Figure 1A). This disorder in the apatite structure is produced by defects created in the apatite structure due to the replacement of phosphate ions (PO_4_^3−^) by carbonates (CO_3_^2−^). Fourier‐transform infrared spectroscopy (FTIR) further supported this observation by identifying vibrational bands in the 420–550 cm^−1^ region, which are characteristic of carbonate ions (CO₃^2−^) substituting phosphate groups in the HA lattice (Figure 1B). Elemental chemical analysis confirmed the presence of 0.83 mol% CO₃^2−^ in the HA structure (Figure 1C). As reported in the literature, the replacement of phosphate groups with carbonate ions in the HA structure typically leads to an increase in the Ca/P ratio to values greater than that of stoichiometric apatite (Ca/P = 1.67), in addition to a reduction in the particle size ratio and an increase in the specific surface area. Accordingly, the BET analysis yielded a specific surface area of 72 m^2^/g.

### 3.2 ATP Quantification Assay Reliably Assessed nCHA Cytotoxicity in ASC Monolayers

**FIGURE 2:**
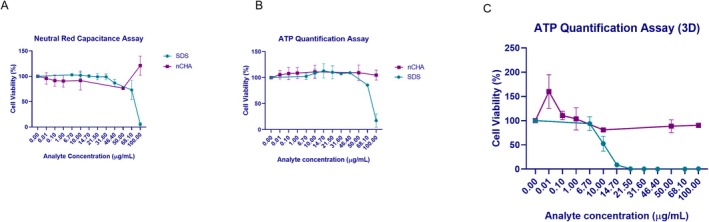
The dose‐dependent curves of ASC exposed to SDS (positive control) and nCHA demonstrate that NPs do not affect cell viability. The ASCs cells were exposed to different concentrations of nCHA or SDS for 48 h and cell viability was analyzed using three different assays. (A, B) Monolayer. (A) Cell viability measured by Neutral Red assay. (B) Cell viability measured by ATP quantification assay. (C) Cell viability of spheroids measured by ATP quantification assay. Spheroid viability was not altered in the presence of nCHA NPs but decreased in the presence of SDS concentrations when the viability was analyzed through ATP quantification. The “0” concentration corresponds to the control group (without nCHA). Data represent three independent biological replicates (*n* = 3), each calculated as the mean of six technical wells. Normality was assessed with the Shapiro–Wilk test (SDS: *p* = 0.0001 in (A) and (B), *p* = 0.0015 in (C); nCHA: *p* = 0.2911 in (A), *p* = 0.6202 in (B), *p* = 0.5331 in (C). Comparisons were performed using the Mann–Whitney U test (unpaired, two‐tailed). No significant differences were detected between cells cultured with or without nCHA in monolayer assays (A: *p* = 0.8371; B: *p* = 0.6065). In spheroids (C), a significant difference was observed (*p* = 0.0079), mainly driven by the lowest concentration of nCHA (0.01 μg/mL), which showed high variability.

NRU assay showed an unusual increase in absorbance when cell monolayers were exposed to 100‐μg/mL nCHA NPs. On the contrary, the neutral red absorbance decreased when the cells were treated with concentrations higher than 40 μg/mL of SDS (Figure 2A). The ATP quantification assay showed a decrease in cell viability when the cells were exposed to SDS concentrations higher than 46,4 μg/mL. In contrast, cell viability remained stable, as expected, when cells were exposed to nCHA (Figure 2B). Overall, the two assays indicated the absence of cytotoxicity of nCHA NPs in the ASC monolayer. Experiments were performed in three independent biological replicates (*n* = 3), each calculated as the arithmetic mean of six technical wells per concentration. For statistical analysis, biological means were used as the unit of replication. Normality was assessed with the Shapiro–Wilk test. SDS‐treated groups significantly deviated from a Gaussian distribution in all assays (neutral red: *p* = 0.0001; ATP 2D: *p* = 0.0001), whereas groups exposed to nCHA were compatible with normality (neutral red: *p* = 0.2911; ATP 2D: *p* = 0.6202). Comparisons were conducted using the non‐parametric Mann–Whitney U test (unpaired, two‐tailed). No significant differences in viability were observed between cells cultured with or without nCHA in monolayer assays (neutral red: *p* = 0.8371; ATP: *p* = 0.6065). Overall, the data support that nCHA did not induce cytotoxicity under the tested conditions and concentrations.

### 3.3 ATP Quantification Assay Consistently Assessed nCHA Cytotoxicity in ASC Spheroids

Due to the unexpected increase in cell viability observed in the NRU uptake assay with ASCs monolayers exposed to nCHA and the difficulty in accessing dyes within the spheroids, the commercial ATP kit, specifically developed for 3D cultures, was chosen to assess the viability of ASC spheroids. The ATP quantification curve (Figure 2C) corroborated the results obtained with the ASC monolayer (Figure 2B). Overall, both assays confirmed the absence of cytotoxicity of nCHA NPs in the ASC spheroids. Experiments were performed in three independent biological replicates (*n* = 3), each calculated as the arithmetic mean of six technical wells per concentration. For statistical analysis, biological means were used as the unit of replication. Normality was assessed with the Shapiro–Wilk test. SDS‐treated groups significantly deviated from a Gaussian distribution in all assays (ATP 3D: *p* = 0.0015), whereas groups exposed to nCHA were compatible with normality (ATP 3D: *p* = 0.5331). Comparisons were conducted using the non‐parametric Mann–Whitney U test (unpaired, two‐tailed). A significant difference was observed between spheroids cultured with and without nCHA (*p* = 0.0079). This difference was mainly driven by the lowest tested concentration of nCHA (0.01 μg/mL), where mean viability exceeded 100% but displayed high variability among replicates. At higher concentrations (1–100 μg/mL), nCHA‐treated spheroids exhibited viability comparable to that of the control. Overall, the data support that nCHA did not induce cytotoxicity under the tested conditions and concentrations.

### 3.4 ASC Spheroids may Be More Sensitive Than Monolayer

**FIGURE 3:**
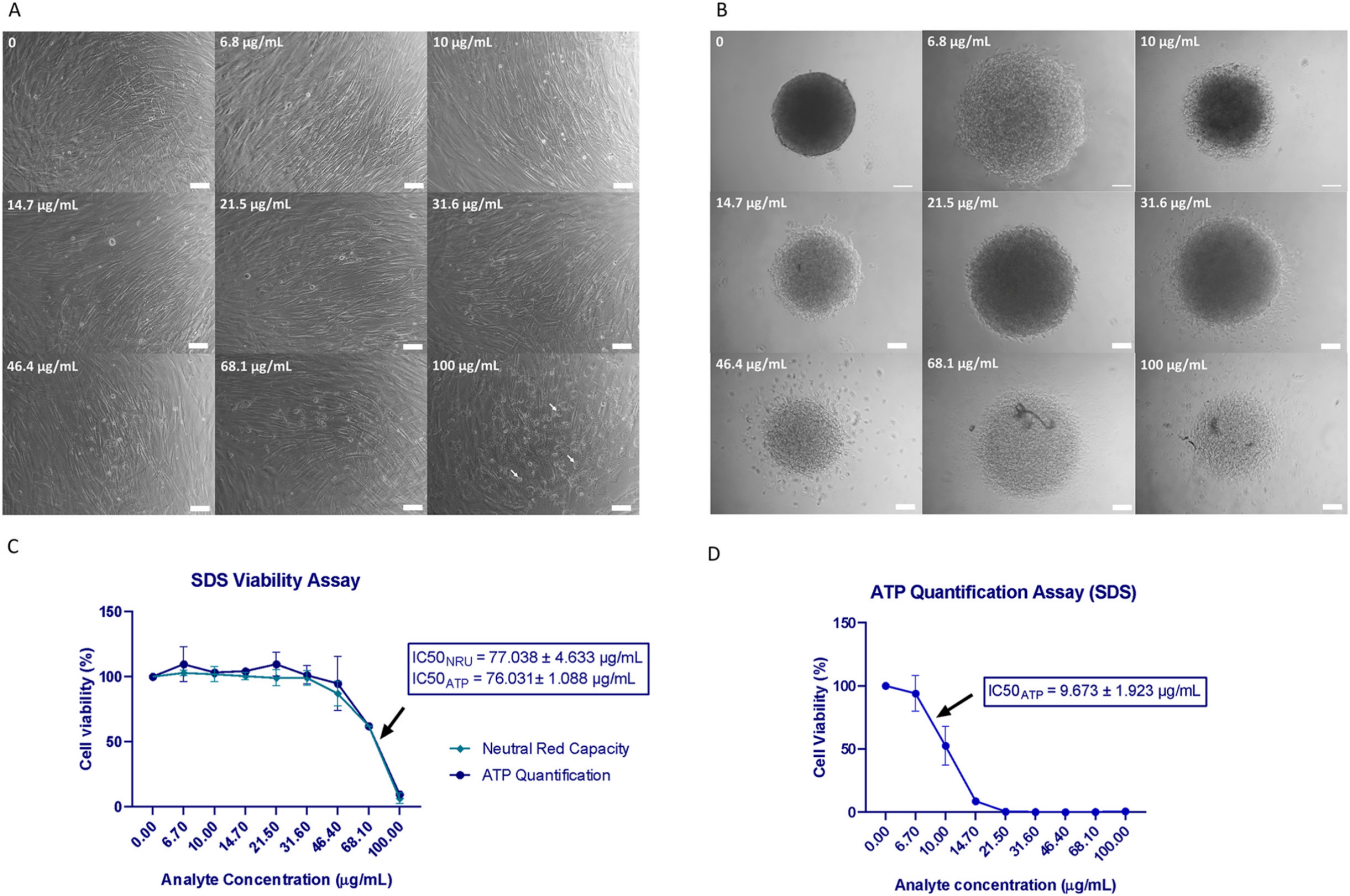
Spheroids are more sensitive to SDS treatment compared to monolayers, showing cells detaching and a lower IC50. (A, B) Phase‐contrast microscopy images of ASC monolayers (A) and ASC spheroids (B) treated with different concentrations of SDS. (A) Arrows indicate rounded cells detaching from monolayer. Scale bar of 100 μm. IC50 of SDS in ASC monolayers (C) and ASC spheroids (D). (C, D) Nonlinear regression curves (four‐parameter logistic model) of cell viability assays used to estimate IC₅₀. Estimated values were IC₅₀_NRU (2D) = 77.0 μg/mL (95% CI: 65.7–88.4; *R* ^2^ = 0.979), IC₅₀_ATP (2D) = 76.0 μg/mL (95% CI: 73.4–78.7; *R* ^2^ = 0.895), and IC₅₀_ATP (3D) = 9.67 μg/mL (95% CI: 6.82–13.7; *R* ^2^ = 0.979). Comparison of IC₅₀ values between neutral red and ATP assays in 2D monolayers showed no significant difference (extra‐sum‐of‐squares *F* test, *p* = 0.387). In contrast, spheroids were significantly more sensitive to SDS than monolayers, as indicated by their markedly lower IC₅₀. The “0” concentration corresponds to the untreated control group. Data represent three independent experiments performed in sextuplicate.

While nCHA was not toxic either for ASC monolayer nor for spheroids, SDS showed a dose‐dependent cytotoxicity in both of them. ASC spheroids were more susceptible to cell death than monolayers, as observed in phase‐contrast images (Figure 3A,B). The unpacking of ASC spheroids is notable even in the lowest SDS concentration (6 μg/mL) and more evident from 46 μg/mL (Figure 3B). Although the cell viability curves were similar between monolayers (Figure 3C) and spheroids (Figure 3D), the IC50 calculation revealed that the concentration‐dependent cytotoxic effect of SDS was distinct between them. ASC spheroids were eight times more sensitive to SDS (IC 50 = 9673 μg/mL—ATP quantification) than the monolayer (IC 50 = 76,031 μg/mL—ATP quantification and IC 50 = 77,038 μg/mL—NRU quantification).

### 3.5 nCHA NPs Treatment Does Not Affect ASC Spheroid Size or Morphology

**FIGURE 4:**
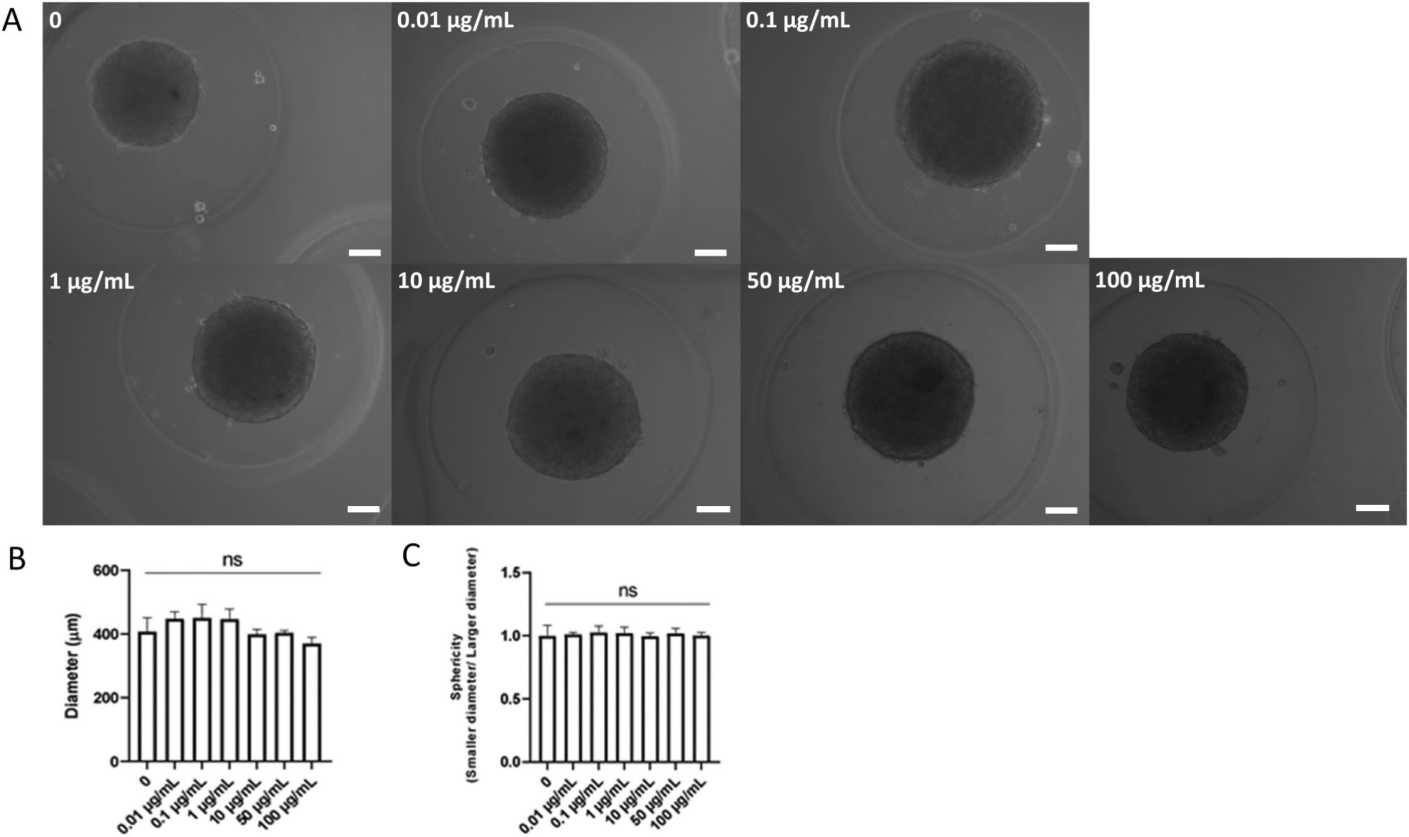
nCHA NP did not affect ASC spheroid morphology. (A) Phase‐contrast microscopy images of ASC spheroids and ASC spheroids diameter (B) and sphericity (C) in the presence of different concentrations of nCHA NP. The “0” concentration corresponds to the control group (without nCHA). Scale bar of 100 μm. Data are presented as mean ± SD from three independent experiments (*n* = 3). For spheroid diameter, data were normally distributed (Shapiro–Wilk, all *p* > 0.05) with homogeneous variances (Brown–Forsythe, *p* = 0.173); one‐way ANOVA indicated a significant overall effect (*F* [6,53] = 10.53, *p* < 0.0001), but Tukey's post hoc test revealed no significant pairwise differences (all ns, *p* > 0.05). For spheroid sphericity, normality was not satisfied across groups, and the Kruskal–Wallis test showed no significant differences (*H* = 3.728, *p* = 0.7134).

Since nCHA NPs did not show cytotoxicity, the next step was to assess their impact on ASC spheroid morphology. One of the first indicators of spheroid viability and stability is their diameter and sphericity (Zanoni et al. 2016). ASC spheroids formed in the presence of nCHA showed a well‐rounded and stable morphology (Figure 4A) and subtle differences in their diameters. Both the spheroids' diameter and sphericity remained similar in the presence of increasing concentrations of nCHA without statistically significant difference (Figure 4B,C). For diameter, data were normally distributed (Shapiro–Wilk, all *p* > 0.05) and variances were homogeneous (Brown–Forsythe, *p* = 0.173). One‐way ANOVA revealed a significant global effect of treatment (*F* [6,53] = 10.53, *p* < 0.0001); however, Tukey's post hoc test indicated no significant pairwise differences compared to the control (all ns, *p* > 0.05). For sphericity, some groups failed the normality test, and thus, a non‐parametric Kruskal–Wallis test was applied, which revealed no significant differences between concentrations (*H* = 3.728, *p* = 0.7134). Together, these results indicate that nCHA exposure did not alter spheroid morphology under the tested conditions.

### 3.6 nCHA NPs Are Predominantly Localized in the Extracellular Matrix of ASC Spheroids

**FIGURE 5:**
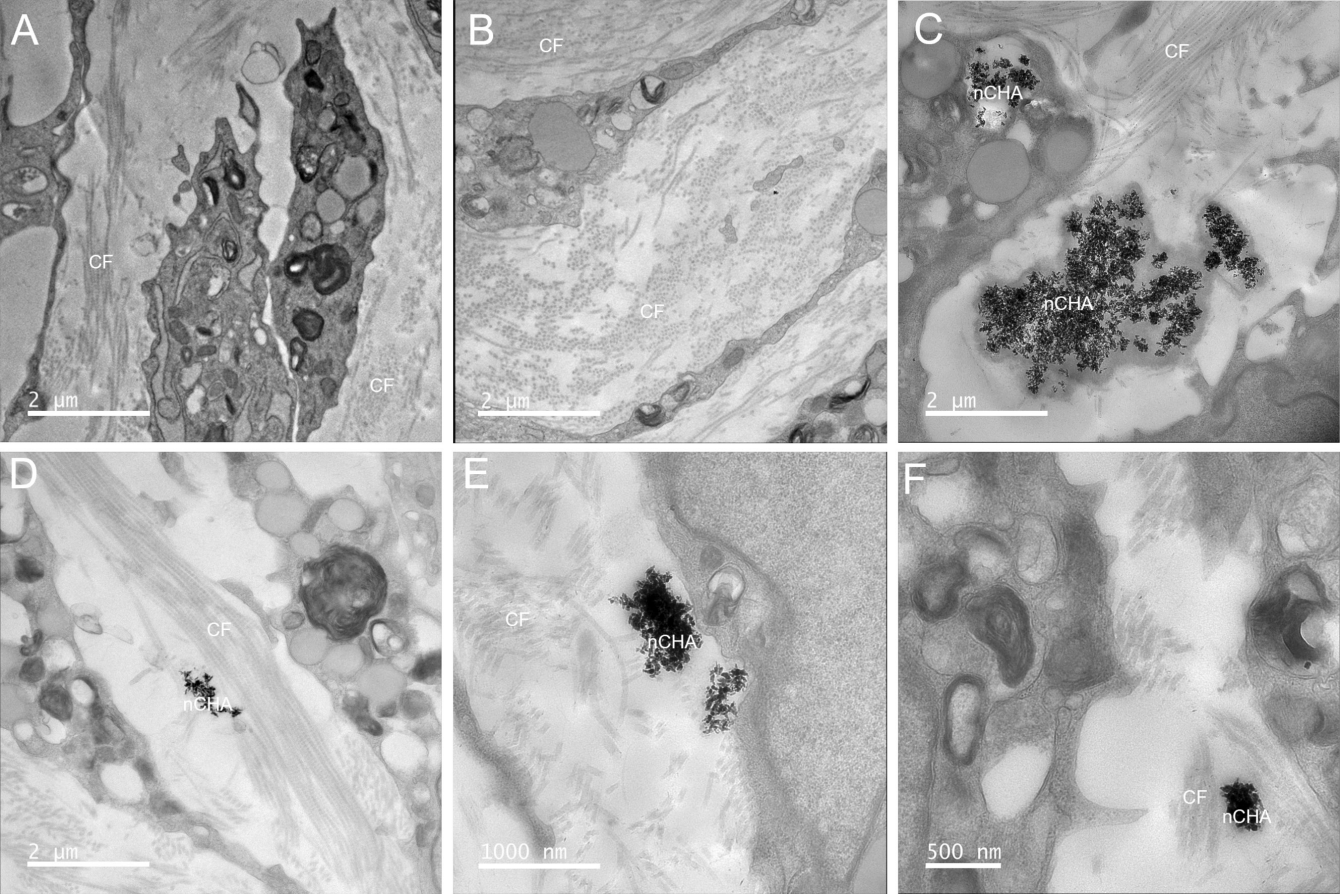
nCHA NP are mainly located in the extracellular matrix of ASC spheroids. Transmission electron microscopy (TEM) images of ASC spheroids in the absence (A, B) and in the presence (C–F) of nCHA at a concentration of 50 μg/mL. Images revealed an abundance of collagen fibers (CF), in particular in the absence of nCHA (A, B). The nCHA are preferably located near collagen fibers among cells (C–F).

Transmission electron microscopy analysis revealed abundant collagen fibers within ASC spheroids. In spheroids cultured in the presence of nCHA (50 μg/mL), nanoparticles were predominantly localized in the extracellular matrix, frequently observed between adjacent cells and in close proximity to collagen fibers (Figure 5A–F). No evident intracellular accumulation of nCHA was detected under the conditions analyzed.

## 4 Discussion

Toxicological assessments are important to identify the hazard or confirm the lack of toxicity of chemicals, drugs, and even biomaterials. The Organization for Economic Cooperation and Development (OECD) validates and accepts some alternative in vitro methods to evaluate toxicity parameters such as irritation, corrosion, and genotoxicity (Hardwick et al. 2020; Gordon et al. 2015). However, the adoption of such methods for nanotoxicology remains limited, primarily due to challenges related to standardization and validation. Although a few standardized protocols have been proposed (ISO 2018; ISO 2017), they still require further adaptation to be broadly implemented.

3D spheroids mimic the in vivo microenvironment, which is why there is increasing interest in their use in toxicology assays as a new approach methodology in alternative methods to animal testing (Fäs et al. 2025). However, few studies have focused on standardizing toxicological analyses in spheroids according to existing international guidelines. Recently, a study was published proposing a standardized approach for genotoxicity assessment in spheroid models (Honza et al. 2024). Therefore, viability assessment methods for 3D spheroids need to be evaluated and chosen with caution due to challenges in the penetration of dyes and possible signal interference from the spheroids. This challenge is also common when working with nanoparticles, adding to other obstacles such as synthesis, aggregation, and biological evaluations, among others (Altammar 2023).

In this study, three viability assays were compared to determine their efficacy and suitability for toxicity assessment in 2D and 3D cell culture models. A known cytotoxic agent, sodium dodecyl sulfate (SDS), and the nCHA, considered biocompatible due to its chemical similarity to calcified biological tissue, were used (Kavasi et al. 2021). nCHA has the additional advantage of enhanced resorption, bioactivity, and osteoconductivity compared to stoichiometric hydroxyapatite (HA) (Calasans‐Maia et al. 2015).

The neutral red uptake (NRU) assay assesses cytotoxicity by measuring the reduction in dye absorption after chemical exposure, providing insights into cellular integrity and acute toxicity. However, it only measures basal cytotoxicity and does not account for selective toxicity or effects on specific cellular functions (ECHA 2024). This method has proven itself hard to use with 3D cultures due to the difficulty in accessing dyes within the spheroids besides the unexpected increase in the signal at the higher concentration of nCHA, which may be due to some interference from the NP (Mello et al. 2020; Andraos et al. 2020).

The most advanced and sensitive method for testing viability in 3D systems has been based on intracellular ATP quantification (Kijanska and Kelm 2004; Idrees et al. 2018). The optimization of lysis conditions led to the development of ATP assays suitable for 3D cell cultures (Messner et al. 2013; Rimann et al. 2014; Fey and Wrzesinski 2012). Bioluminescent ATP detection assays are robust, sensitive, and scalable for high‐throughput screening, unlike traditional colorimetric methods, such as the resazurin reduction (Alamar blue) or tetrazolium (MTT) assays, which are not suitable for spheroids (Idrees et al. 2018; Bonnier et al. 2015; Walzl et al. 2014).

An ATP quantification assay kit available for both 2D and 3D cell culture was used to compare the cytotoxicity effect of SDS. The IC₅₀ values obtained from SDS dose–response curves were similar between the ATP quantification and NRU assays in 2D cultures (IC₅₀NRU = 77.038 μg/mL and IC₅₀ATP = 76.031 μg/mL), confirming the reliability of both methods for monolayer assessments. In contrast, the IC₅₀ for SDS in ASC spheroids was markedly lower (9.67 μg/mL) than the monolayer, representing an approximately eight times lower than that observed in monolayers. This enhanced responsiveness aligns with the concentration‐dependent disaggregation of spheroids in the presence of SDS, supporting the unpacking of spheroid structures observed in phase contrast images, and further underscores the superior sensitivity of 3D microtissues as new approach methodologies for nanotoxicological evaluation. Welch and collaborators evaluated the responsiveness of a human airway 3D model to SDS, a cytotoxic agent, following international guidelines for acute inhalation toxicity. The authors reported that SDS caused extensive damage to cell morphology (Welch et al. 2021). To date, no published study in the scientific literature using spheroids has applied the SDS dose–response curve as recommended by international guidelines.

As expected, the nCHA NP did not show toxicity either in the monolayer nor in the spheroids. This result highlights the suitability of the 3D spheroid model to be used in toxicological assessment of nanoparticles as well as the potential to explore the functionalization of spheroids aiming tissue engineering applications. Since nCHA nanoparticles were located in the extracellular matrix of ASC spheroids, particularly in regions enriched in collagen, their localization may meaningfully influence early osteogenic events. Collagen fibrils act as structural templates that organize calcium phosphate precursors and guide their transformation into ordered apatite, a process that resembles the hierarchical architecture of native bone (Srinivasan et al. 2015). Although the extracellular matrix alone does not initiate mineralization, the coordinated activity of non‐collagenous proteins embedded within it concentrates calcium and phosphate ions, facilitating the nucleation and growth of hydroxyapatite at specific sites. The proximity of nCHA to collagen‐rich domains may therefore stabilize mineral precursors, promote nucleation and crystal alignment, and contribute to the establishment of an osteoconductive microenvironment (Zhou and Lee 2011). This phenomenon suggests that nCHA‐functionalized spheroids may support controlled extracellular mineral deposition and potentially contribute to the development of mature bone‐like tissue in vitro for future applications in bone tissue engineering—an aspect currently under investigation by our research group.

Among the limitations of our study is the use of ASCs from a single donor. Although this approach is consistent with international guidelines for in vitro cytotoxicity testing, including OECD TG 129 and TG 432 as well as ICCVAM, ECVAM, and FDA recommendations, which emphasize assay reproducibility, methodological consistency, and the use of validated analytical endpoints rather than the inclusion of multiple biological donors, it remains possible that inter‐donor variability may influence certain phenotypic and functional properties of mesenchymal stem cells. Thus, incorporating cells from multiple donors in future studies will be important to expand the generalizability of the model and to better characterize biological variability relevant to translational and regulatory applications.

Additionally, cell viability was assessed only in the short term, whereas for tissue engineering applications, it is essential to evaluate long‐term cell viability, especially considering that commonly employed differentiation assays require extended culture periods. Future studies should include extended culture periods to assess potential delayed cytotoxic effects and to ensure the long‐term compatibility of the spheroid‐nCHA system.

## 5 Conclusion

In this study, the suitability of human ASC spheroids for toxicological testing using nCHA NP, known for their biocompatibility, was demonstrated. Moreover, the use of an ATP quantification assay for assessing spheroid viability was shown to be appropriate. While other nanoparticles should be tested to further validate the model, the feasibility of producing a 3D in vitro new approach methodology (NAM) for toxicological testing was demonstrated. Additionally, the integration of nCHA NP within the extracellular matrix of spheroids was achieved, paving the way for future applications of functionalized spheroids in bone engineering.

## Author Contributions

L.S.B. and A.M.R. were involved in the conception of study design. L.S.B. and A.M.R. contributed to the funding acquisition. L.S.B. and B.M. contributed to the design and realization of experiments, data acquisition, analysis, and interpretation. L.S.B., B.M., and M.G.P. were involved in manuscript preparation. R.M.G. and I.A. performed experiments. L.S.B. and A.M.R/ were involved in revising and editing the manuscript. All authors read and approved the final manuscript.

## Funding

This study was supported by the National Council for Scientific and Technological Development (CNPq) (Finance Code: 309289/2022‐0), Carlos Chagas Filho Foundation for Research Support of the State of Rio de Janeiro (Faperj) (Finance Code: E‐26/204.291/2024), Rede NanoSaude: E‐26/010.000981/2019, and the Office of Naval Research (ONR) (Finance Code: N62909–21‐1‐2091). The authors also would like to thank the Coordenação de Aperfeiçoamento de Pessoal de Nível Superior, Brazil (CAPES) (Finance Code: 8888.724389/2022‐00).

## Conflicts of Interest

The authors declare no conflicts of interest.

## Data Availability

The data that support the findings of this study are available from the corresponding author upon reasonable request.
